# Feeding incremental amounts of ground flaxseed: effects on diversity and relative abundance of ruminal microbiota and enteric methane emissions in lactating dairy cows

**DOI:** 10.1093/tas/txad050

**Published:** 2023-05-29

**Authors:** Kleves V Almeida, Tales L Resende, Luiz Henrique P Silva, Christopher D Dorich, Andre B D Pereira, Kathy J Soder, Andre F Brito

**Affiliations:** Department of Agriculture, Nutrition, and Food Systems, University of New Hampshire, Durham, NH 03824, USA; Escola de Veterinária, Universidade Federal de Minas Gerais, Belo Horizonte, MG 30161, Brazil; Department of Agriculture and Food Science, Western Kentucky University, Bowling Green, KY 42101, USA; Institute for the Study of Earth, Oceans, and Space and Department of Earth Sciences, University of New Hampshire, Durham, NH 03824, USA; Department of Agriculture, Nutrition, and Food Systems, University of New Hampshire, Durham, NH 03824, USA; Pasture Systems and Watershed Management Research Unit, USDA-Agricultural Research Service, University Park, PA 16802, USA; Department of Agriculture, Nutrition, and Food Systems, University of New Hampshire, Durham, NH 03824, USA

**Keywords:** archaea, methanogenesis, microbiome, oilseed

## Abstract

We evaluated the effects of incremental amounts of ground flaxseed (**GFX**) on diversity and relative abundance of ruminal microbiota taxa, enteric methane (**CH**_**4**_) emissions, and urinary excretion of purine derivatives (**PD**) in lactating dairy cows in a replicated 4 × 4 Latin square design. Twenty mid-lactation Jersey cows were used in the study. Of these 20 cows, 12 were used for ruminal sampling, 16 for enteric CH_4_ measurements, and all for spot urine collection. Each period lasted 21 d with 14 d for diet adaptation and 7 d for data and sample collection. Diets were formulated by replacing corn meal and soybean meal with 0%, 5%, 10%, and 15% of GFX in the diet’s dry matter. Ruminal fluid samples obtained via stomach tubing were used for DNA extraction. Enteric CH_4_ production was measured using the sulfur hexafluoride tracer technique. Diets had no effect on ruminal microbiota diversity. Similarly, the relative abundance of ruminal archaea genera was not affected by diets. In contrast, GFX decreased or increased linearly the relative abundance of Firmicutes (*P* < 0.01) and Bacteroidetes (*P* < 0.01), respectively. The relative abundance of the ruminal bacteria *Ruminococcus* (*P* < 0.01) and *Clostridium* (*P* < 0.01) decreased linearly, and that of *Prevotella* (*P* < 0.01) and *Pseudobutyrivibrio* (*P* < 0.01) increased linearly with feeding GFX. A tendency for a linear reduction (*P* = 0.055) in enteric CH_4_ production (from 304 to 256 g/d) was observed in cows fed increasing amounts of GFX. However, neither CH_4_ yield nor CH_4_ intensity was affected by treatments. Diets had no effect on the urinary excretion of uric acid, allantoin, and total PD. Overall, feeding GFX decreased linearly the relative abundance of the ruminal bacterial genera *Ruminococcus* and *Clostridium* and enteric CH_4_ production, but no change was seen for CH_4_ yield and CH_4_ intensity, or urinary excretion of total PD, suggesting no detrimental effect of GFX on microbial protein synthesis in the rumen.

## Introduction

We reported in our companion paper (Resende et al., 2015) that the milk proportions of total n-3 fatty acids and α-linolenic acid (**ALA**) increased linearly in dairy cows fed diets containing incremental amounts (0%, 5%, 10%, and 15%; dry matter [**DM**] basis) of ground flaxseed (**GFX**; *Linum usitatissimum*). It is also important to note that polyunsaturated fatty acids (**PUFA**) present in oilseeds such as GFX are toxic to ruminal methanogens and protozoa (Patra, 2013), which can decrease enteric methane (**CH**_**4**_) production in lactating dairy cows. Martin et al. (2016) observed linear reductions in CH_4_ production, CH_4_ yield, and CH_4_ intensity in dairy cows fed hay- or corn silage-based diets supplemented with increasing levels of extruded flaxseed (0%, 5%, 10%, and 15%; DM basis). However, there is limited research investigating the effects of GFX on ruminal microbiota diversity and taxa and enteric CH_4_ emissions in lactating dairy cows fed high-forage diets. Schroeder et al. (2014) showed that the in situ DM degradability of GFX, rolled flaxseed, and whole flaxseed averaged 11.3%, 4.5%, and 0.57% per hour, respectively. These results suggest that the processing method used and form of flaxseed fed likely have different effects on ruminal fermentation processes including methanogenesis, thus requiring further research with GFX.

Huang et al. (2021) observed that dairy cows fed GFX (6.38%; DM basis) had the lowest and greatest relative abundance of ruminal bacterial belonging to the Bacteroidetes and Firmicutes phyla, respectively, compared with the control and whole flaxseed diets. They further reported that feeding GFX decreased the ruminal molar proportion of acetate, and increased that of propionate and butyrate, thus in line with shifts detected for Bacteroidetes and Firmicutes. Deusch et al. (2017) demonstrated that Firmicutes species are primarily responsible for enzymes involved in butyrate and formic acid production, while Bacteroidetes are mostly associated with enzymes related to propionate and acetate synthesis in the rumen. Therefore, changes in ruminal microbiota and fermentation profile in cows fed GFX (Deusch et al.; 2017; Huang et al., 2021) suggest that methanogenesis may be also affected. For instance, CH_4_ production decreased linearly in dual-flow continuous culture fermentors dosed with incremental amounts of GFX (0%, 5%, 10%, and 15%; DM basis), which coincided with a linear increase in the molar proportion of propionate (Soder et al., 2012). In our companion paper (Resende et al., 2015), whereas DM intake (**DMI**) decreased linearly in cows fed GFX, the ruminal molar proportion of propionate increased linearly, with these responses commonly associated with a reduction in enteric CH_4_ production. According to the United States Environmental Protection Agency (US EPA, 2021), 27% of all CH_4_ emissions in the country originate primarily from cattle enteric fermentation, and are behind only to natural gas and petroleum systems that together contribute 30% of the total. In addition to being a potent greenhouse gas, up to 10% of the dietary gross energy intake can be lost through enteric CH_4_ production in lactating dairy cows (Niu et al., 2018). Therefore, strategies to mitigate ruminal methanogenesis, particularly in high-forage diets, are needed to reduce the environmental impact of dairy systems while improving energy use efficiency in lactating dairy cows.

We hypothesized that enteric CH_4_ production would be reduced linearly in response to incremental amounts of GFX due to the toxic effects of GFX-PUFA on the growth of methanogens and cellulolytic bacteria. This will be inferred by changes in the relative abundance of ruminal microbiota taxa and the urinary excretion of purine derivatives (**PD**), which is commonly used as a proxy for microbial growth in the rumen that can be negatively affected by PUFA supply from GFX. Our objective was to evaluate the effects of increasing levels of GFX on diversity and relative abundance of ruminal microbiota taxa, enteric CH_4_ emissions, and urinary excretion of PD in lactating dairy cows.

## Material and Methods

Experimental procedures involving cows were approved by the University of New Hampshire (Durham) Institutional Animal Care and Use Committee (protocol n. 110605). Specific details regarding feeding and management of cows, as well as the ingredient and chemical composition of the experimental diets and sample collection (i.e., feeds, milk, blood, and feces) and analyses are reported in our companion paper (Resende et al., 2015).

### Cows, Experimental Design, and Treatments

Twelve multiparous (112 ± 68 days in milk and 441 ± 21 kg of body weight) and eight primiparous (98 ± 43 days in milk and 401 ± 43 kg of body weight) organic-certified Jersey cows were selected to participate in the experiment. Of these 20 cows, 12 (8 multiparous and 4 primiparous) were used for ruminal fluid sampling, 16 (8 multiparous and 8 primiparous) for enteric CH_4_ measurements, and all 20 for spot urine collection. Animals were housed in a bedded-pack barn with dried pine shavings as bedding. The bedding area (132 m^2^) opens to a concrete-floor outdoor lot (478 m^2^) in compliance with the USDA National Organic Program livestock living condition regulations (USDA AMS, 2010) that mandate year-round access to the outdoors for all ruminants.

Cows were blocked by days in milk or parity and, within block, randomly assigned to treatment sequences in a replicated 4 × 4 Latin square design, with squares balanced for potential first-order carryover effects in subsequent periods. Each period lasted 21 d, with 14 d for diet adaptation and 7 d for data and sample collection. Cows were fed twice daily (~9000 and 1600 h) using the electronic recognition Calan doors system (American Calan Inc., Northwood, NH) to individualize treatment intake. Diets were offered as total mixed rations (**TMR**; 63:37 forage-to-concentrate ratio) with corn meal and soybean meal replaced by incremental levels (0%, 5%, 10%, or 15% of the diet DM) of GFX (AgMotion Specialty Grains Inc., Minneapolis, MN).

### Ruminal Fluid Sampling and Microbiota Analyses

Ruminal fluid samples were collected during days 17 to 19 of each period approximately 7 h after the a.m. feeding via stomach tubing. Samples were then filtered through 4 layers of cheesecloth into volumetric flasks to a final volume of 250 mL and preserved on dry ice until transported to the laboratory. Next, aliquots of 0.6 mL of ruminal fluid from each day were pooled per cow into 2-mL cryovials (1.8 mL total) and stored at −80 °C until shipped in dry ice to MR DNA Laboratory (Shallowater, TX) for DNA extraction and MiSeq sequencing as reported in detail by Silva et al. (2022). In brief, microbial DNA (prokaryotes) was extracted from ruminal fluid samples using the PowerSoil DNA isolation method (kit #12888–100; MO BIO Laboratories Inc., Carlsbad, CA) and quantified via spectrophotometry (2000 UV-VIS, NanoDrop spectrophotometer; Thermo Fischer Scientific, Waltham, MA). Amplification of the V4 variable region of 16S rRNA genes was carried out with the primer pairs 515F (5ʹ- GTGCCAGCMGCCGCGGTAA) and 806R (5ʹ-GGACTACHVHHHTWTCTAAT) as reported previously (Caporaso et al., 2012). The HotStarTaq Plus Master Mix DNA polymerase kit (QIAGEN, Hilden, Germany) was used for PCR amplifications following conditions and elongation steps shown in Silva et al. (2022). Purified PCR products were utilized to prepare a sequencing library following the Illumina TruSeq (Illumina Inc., San Diego, CA) protocol, with sequencing analysis performed on a MiSeq (Illumina Inc.) following the manufacturer’s guidelines.

The 250-bp paired-end raw Illumina reads from ruminal fluid microbiota were initially evaluated using FastQC (v.0.11.4; Babraham Institute, Cambridge, UK). Next, reads were processed to remove Illumina adapters and trimmed to remove low-quality reads (Phred ≤30) using Trimmomatic v.0.33 (Bolger et al., 2014), with high-quality paired-end reads merged using the PEAR software (Zhang et al., 2014). All postprocessed reads were submitted to the QIIME2 package for microbiota genetic quantitative analysis (Bolyen et al., 2019). Amplicon errors from the high-quality reads were initially corrected using DADA2 (Callahan et al., 2016) followed by the QIIME2 “demux” function to remove chimeras and duplicated sequences, and to demultiplex the reads based on representative sequences. Operational taxonomic units (**OTU**) were defined by clustering at 3% divergence (97% similarity). Final OTU were taxonomically classified using BLASTn against a curated database (DeSantis et al., 2006). Bacterial and archaeal genera were considered for statistical analysis when relative abundance was greater than 0.1% and 0.01%, respectively. Diversity of ruminal microbiota (prokaryotes) in response to diets was evaluated using the α-diversity indices OTU, Shannon, and Faith. The β-diversity principal coordinate analysis (**PCoA**) was performed using the Bray–Curtis method for unweighted (qualitative) UniFrac distances for each treatment as detailed in Silva et al. (2022).

### Enteric CH_4_ Emission Measurements

Enteric CH_4_ production was measured during the last 5 d of each period (days 17 to 21 of each period) using the sulfur hexafluoride (**SF**_**6**_) tracer technique originally developed by Johnson et al. (1994), but with adaptations in the permeation tubes as reported by Dorich et al. (2015). Permeation tubes were weighed for 6 wk to determine SF_6_ permeation rate, which averaged 1.47 ± 0.12 mg/d. Polyvinyl chloride canisters (10.2 cm diameter, 17.8 cm length, and 1 L volume) fitted with caps attached to ball shutoff valves connected to the sampling apparatus were fixed to the back of the cows using back-mounted harnesses. Canisters were removed twice daily before each milking (0600 h and 1500 h) and replaced (~1 h after milking) with new flushed and evacuated canisters. Two canisters were placed near the feed bunk and bedding area to account for background gas concentrations in the calculations of gaseous emissions. Gaseous samples were collected via syringes, with gases injected into pre-evacuated 10-mL vials with septa and stored until analysis using a Shimadzu GC-8A (Shimadzu Corp., Kyoto, Japan) equipped with flame ionization detection and electron capture detection for CH_4_ and SF_6_, respectively. A data filter with CH_4_ production ranging from 79 to 729 g/d based on individual cow data (Niu et al., 2018) was applied to our data set to discard extreme and impossible (i.e., negative emissions) CH_4_ values. Calculations of enteric CH_4_ production were done as described by Johnson et al. (1994).

### Urinary Sampling and Analyses

Spot samples of urine were collected once daily for 5 consecutive days (days 17 to 21 of each period) at 0000 h, 0800 h, 1200 h, 1600 h, and 2000 h by stimulation of the pudendal nerve massaging the area below the vulva or during voluntary urination. After collection, 8 mL of urine was acidified with 400 µL of 6 N HCl per time point and pooled over 5 d to generate 1 sample per cow for each period. Urinary samples were stored at −20 °C in 50-mL centrifuge tubes until analyzed for total N, creatinine, allantoin, and uric acid. Concentration of total N in urine was assessed by micro-Kjeldahl analysis (Dairy One Forage Testing Laboratory, Ithaca, NY). Creatinine concentration was measured colorimetrically using a commercially available kit (Cayman Chemical Co., Ann Arbor, MI) with a chromate microplate reader set at a wavelength of 492 nm (Awareness Technology Inc., Westport, CT). Allantoin concentration was determined as reported by Chen et al. (1992), and that of uric acid using the Stanbio Uric Acid LiquiColor kit (procedure #1045; Stanbio Laboratory, Boerne, TX). Urinary allantoin and uric acid concentrations were read at wavelengths of 522 and 520 nm, respectively, on a UV-visible spectrophotometer (Beckman Coulter Inc., Brea, CA). Urinary volume was estimated based on the concentration of creatinine assuming a constant creatinine excretion rate of 0.212 mmol/kg of BW (Chizzotti et al., 2008). Urinary excretion of total N, allantoin, uric acid, and total PD (allantoin + uric acid) was calculated by multiplying the concentration of each of these metabolites by the urinary volume.

### Statistical Analyses

Normality of residuals and homogeneity of variance were examined for each variable by the Shapiro–Wilk test using the UNIVARIATE procedure of SAS (version 9.4; SAS Institute Inc.). Data were analyzed using the MIXED procedure of SAS according to a replicated 4 × 4 Latin square design, with treatment, period, and square entered as fixed effects, and cow within square treated as a random effect. Treatment × square interactions were removed from the final model when *P* ≥ 0.25. Orthogonal polynomial contrasts (linear, quadratic, and cubic) were used to test the effects of incremental dietary levels of GFX on variable responses. Significance was declared at *P* ≤ 0.05 and tendencies at 0.05 < *P* ≤ 0.10.

## Results and Discussion

### Diversity and Relative Abundance of Ruminal Microbiota Taxa

A total of 2,852,037 high-quality sequences were generated in the 16S rRNA gene amplicon sequencing from 48 samples. The α-diversity indices OTU, Shannon, and Faith were not affected in dairy cows fed incremental amounts of GFX (Table 1). Similarly, Petri et al. (2018) reported no changes in OTU and Shannon with feeding an extruded flaxseed-based supplement at 25% of the diet DM either incorporated into a TMR or when fed prior to offering hay to crossbred steers. Huang et al. (2021) observed reductions in the α-diversity indices Shannon and Simpson, no changes in Ace, Shao, and Sobs, and an increase in Goods coverage in dairy cows receiving GFX versus control. Overall, flaxseed fed as an extruded (Petri et al., 2018) or as a ground (Huang et al., 2021; present study) form had minimal impact on α-diversity indices, thus suggesting that richness (i.e., number of taxonomic groups) and evenness (i.e., distribution of abundances of the groups) of the ruminal microbiota (prokaryotes) were not affected by flaxseed supplementation.

**Table 1.** Ruminal fluid microbiota diversity and enteric CH_4_ emissions in lactating Jersey cows fed incremental amounts of ground flaxseed (GFX)

Item	GFX, % of the diet DM				SEM	*P*-value^1^		
	0	5	10	15		*L*	*Q*	*C*
α-Diversity indices								
Observed OTU	1,019	996	1,000	986	30.4	0.49	0.88	0.73
Shannon	8.69	8.64	8.58	8.63	0.05	0.34	0.36	0.64
Faith	79.4	77.2	77.9	77.0	1.68	0.37	0.69	0.54
CH_4_ emissions								
CH_4_ production, g/d	304	268	262	256	17.9	0.06	0.36	0.69
CH_4_ yield, g/kg of DMI	18.3	16.3	16.9	15.9	1.15	0.19	0.63	0.36
CH_4_ intensity, g/kg of ECM^2^	12.5	10.8	11.0	11.2	0.82	0.27	0.22	0.60

^1^Probability of linear (*L*), quadratic (*Q*), and cubic (*C*) effect.

^2^Energy-corrected milk = [0.327 × milk yield (kg/d)] + [12.95 × fat yield (kg/d)] + [7.2 × protein yield (kg/d)] (Orth, 1992).

**Figure 1.**
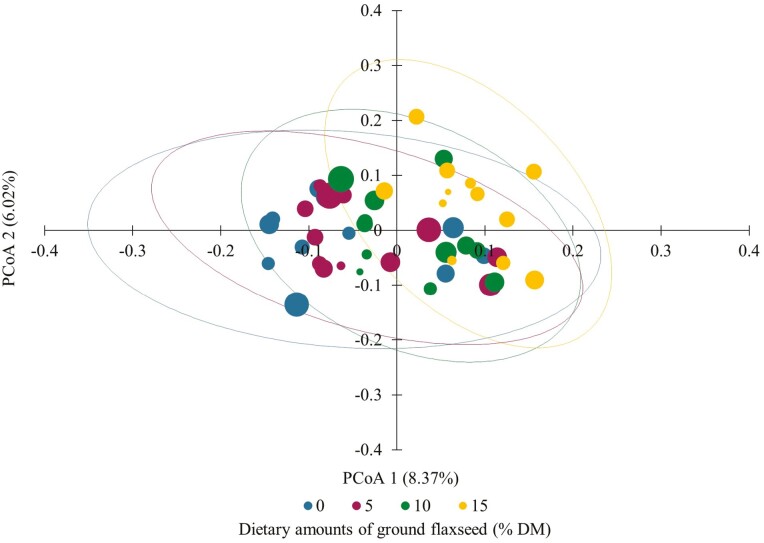
Principal coordinate analysis (PCoA) based on unweighted UniFrac dissimilarities of ruminal fluid microbiota community structures in lactating Jersey cows fed incremental amounts (0%, 5%, 10%, and 15% of the diet DM) of ground flaxseed.

The β-diversity PCoA data showed no overall unifying differences in ruminal microbiota community structures in dairy cows fed incremental dietary levels of GFX as shown by the unweighted UniFrac dissimilarities (Figure 1). These results suggest that diets did not affect the overall composition of ruminal microbiota because community grouping by clusters was not evident. In contrast, Huang et al. (2021) observed a clear separation between diets in the PCoA1 axis, with most control and GFX cows distributed in the negative and positive score values, respectively. However, they were not able to separate control and GFX groups based on values of the PCoA2 axis. Discrepancy in PCoA when comparing Huang et al. (2021) and our study is not straightforward to explain but it may be related to the difference in the forage sources used in the basal diets (corn silage and alfalfa hay vs. mixed-mostly grass baleage and hay) and animal breed (Holstein vs. Jersey). Islam et al. (2021) reported, based on PCoA, the formation of 2 distinct cluster groups suggesting that the overall composition of the ruminal microbiota was different between Holstein and Jersey steers fed the same TMR.

**Figure 2.**
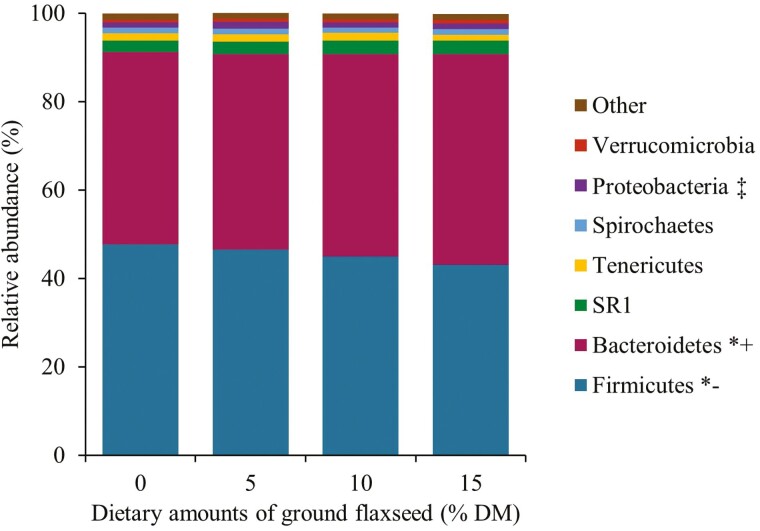
Relative abundance (%) of ruminal bacteria phyla in lactating Jersey cows supplemented with incremental amounts (0%, 5%, 10%, and 15% of the diet DM) of ground flaxseed. *(+) Indicates an increased linear effect, *(−) a decreased linear effect, and ‡ indicates a cubic effect. "Other" represents unassigned operational taxonomic units.

**Figure 3.**
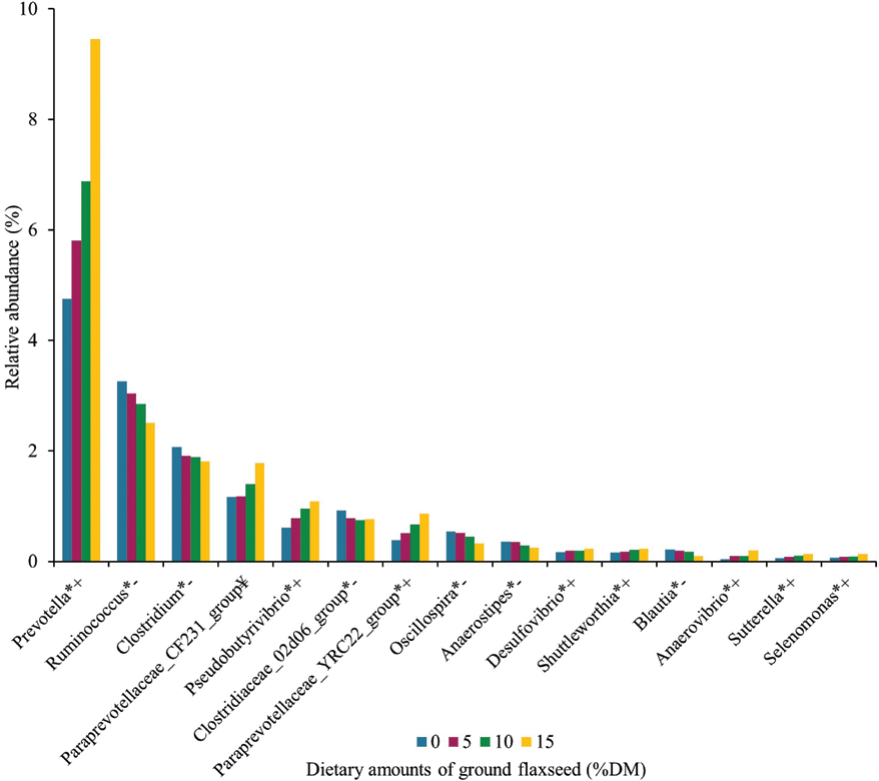
Relative abundance (%) of ruminal bacteria genera (only genus > than 0.1% and *P* < 0.05 are presented) in lactating Jersey cows supplemented with incremental amounts (0%, 5%, 10%, and 15% of the diet DM) of ground flaxseed. *(+) Indicates an increased linear effect, *(−) a decreased linear effect, and ¥ indicates a quadratic effect.

The relative abundance of Firmicutes decreased linearly (*P* < 0.01), whereas that of Bacteroidetes increased linearly (*P* < 0.01) with feeding incremental amounts of GFX (Figure 2). A cubic effect (*P* < 0.01) was observed for the relative abundance of Proteobacteria (Figure 2). However, cubic response is difficult to explain biologically. In contrast, diets did not affect the relative abundance of SR1, Tenericutes, Spirochaetes, and Verrucomicrobia (Figure 2). Previous research showed that PUFA, particularly ALA is toxic to ruminal cellulolytic bacteria (Maia et al., 2007), suggesting that the linear reduction in Firmicutes observed herein may have been associated with ALA toxicity to fibrolytic bacteria (e.g., *Ruminococcus*) belonging to this phylum. The linear increase in the relative abundance of Bacteroidetes in cows fed GFX is in line with that found for *Prevotella* (Figure 3), as the *Prevotella* genus belongs to the Bacteroidetes phylum. It is important to note that *Prevotella* has been implicated in biohydrogenation of PUFA (Huws et al., 2011) and metabolism of flaxseed lignans (Schogor et al., 2014; Brito and Zang, 2019) as discussed in detail below.

We observed linear reductions (*P* < 0.01) in the relative abundance of the ruminal bacteria genera *Ruminococcus, Clostridium, Clostridiaceae_02d06_group, Oscillospira, Anaerostipes,* and *Blautia,* as well as a quadratic decrease (*P* < 0.01) in that of the *Paraprevotellaceae_CF231_group* in cows fed incremental amounts of GFX (Figure 3). Contrarily, the relative abundance of *Prevotella, Pseudobutyrivibrio, Paraprevotellaceae_YRC22_group, Desulfovibrio, Shuttleworthia, Anaerovibrio, Sutterella,* and *Selenomonas* increased linearly (*P* < 0.01) in response to GFX (Figure 3). It is well documented that bacterial species belonging to the *Ruminococcus* genus are primarily cellulolytic (Sijpesteijn, 1951; Terry et al., 2019), with the *Clostridium* and *Blautia* genera also involved in fiber degradation in the rumen (Terry et al., 2019). We observed in our companion paper a linear decrease in the ruminal molar proportion of acetate and linear reduction tendencies for the apparent total-tract digestibilities of neutral detergent fiber and acid detergent fiber with feeding GFX (Resende et al., 2015), thus in line with the decreased relative abundance of *Ruminococcus*, *Clostridium*, and *Blautia*. Yang et al. (2009) demonstrated that compared with cows fed the control diet, those supplemented with soybean oil or flaxseed oil, both at 4% of the diet DM, had decreased counts (cfu/mL) of ruminal cellulolytic bacteria and protozoa, and reduced amount (ng) of DNA copies of *Ruminococcus albus*, *Ruminococcus flavefasciens*, and *Fibrobacter succinogenes*. Maia et al. (2007) showed that ALA was toxic to ruminal cellulolytic bacteria, which may be associated with the linear reduction in the relative abundance of fibrolytic bacteria in cows offered GFX as discussed above.

Increased relative abundance of ruminal *Anaerovibrio*, *Pseudobutyrivibrio*, and *Selenomonas* with feeding increasing dietary levels of GFX (Figure 3) is possibly associated with lipolytic or biohydrogenation activities of bacteria belonging to these 3 genera (Lourenço et al., 2010; Huws et al., 2015). In fact, Huws et al. (2015) showed that the relative abundance of *Pseudobutyrivibrio* was greater in crossbred steers supplemented with flaxseed oil (3% of the diet DM) than those receiving the control diet. Resende et al. (2015) reported that the milk proportions of total *trans*-18:1 FA and total CLA increased linearly in response to GFX, indicating that enhanced supply of ALA led to the accumulation of ruminal biohydrogenation intermediates in milk fat.

We also observed that the relative abundance of ruminal *Prevotella* increased linearly in cows fed diets supplemented with incremental amounts of GFX (Figure 3). The *Prevotella* genus is composed of bacterial species that utilize a variety of nutrients such as protein, starch, pectin, and hemicellulose (Russell, 2002). In our companion paper (Resende et al., 2015), we observed that the dietary concentration of starch decreased from 190 to 115 g/kg of DM and that of total FA increased from 24.9 to 48.1 g/kg of DM by replacing corn meal and soybean meal with GFX, suggesting that elevated supply of ALA may have been associated with the linear increase in the relative abundance of ruminal *Prevotella*. In support, Li et al. (2015) reported greater relative abundance of *Prevotella* in steers fed flaxseed oil than those in the control diet. Further, flaxseed is the richest source of lignans, which are primarily found in the outer fibrous layers of flaxseed (Brito and Zang, 2019). Previous in vitro work using pure cultures of ruminal bacteria revealed that *Prevotella* actively metabolized secoisolariciresinol diglucoside, the most abundant lignan in flaxseed (Schogor et al., 2014).

**Figure 4.**
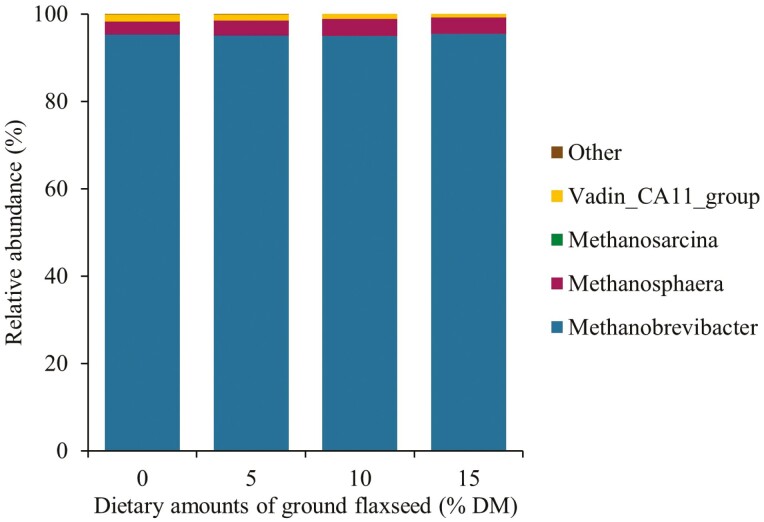
Relative abundance (%) of ruminal archaea genera in lactating Jersey cows supplemented with incremental amounts (0%, 5%, 10%, and 15% of the diet DM) of ground flaxseed. "Other" represents unassigned operational taxonomic units.

There was no effect of GFX on the relative abundance of the ruminal archaea genera (Figure 4). The most abundant ruminal archaea genus was *Methanobrevibacter* followed by *Methanosphaera*, *Vadin_CA11*_group, and *Methanosarcina*. Petri et al. (2018) reported no differences in the relative abundance of ruminal *Methanobrevibacter* in crossbred steers fed an extruded flaxseed-based supplement. They also observed that *Methanobrevibacter* was the most abundant (97%) archaeal genus in the rumen. Supplementation with PUFA has been associated with inhibitory effects on the metabolic activity and numbers of ruminal methanogens (Patra, 2013), which can ultimately affect their relative abundance in the rumen. However, the lack of GFX effect in the relative abundance of the archaea genera in the present study is not clear and difficult to explain with the available data.

### Methane Emissions

Enteric CH_4_ production tended to decrease linearly (*P* = 0.055) with feeding incremental amounts of GFX (Table 1). However, diets did not affect CH_4_ yield and CH_4_ intensity. Resende et al. (2015) reported that DMI decreased linearly in cows fed increasing dietary levels of GFX, thus in line with the current response in CH_4_ production. It is well established the positive linear relationship between DMI and enteric CH_4_ production in lactating dairy cows (Niu et al., 2018). Resende et al. (2015) observed that the ruminal molar proportion of propionate increased linearly, further supporting the reduction in CH_4_ production documented herein as the chemical reactions leading to propionate production in the rumen use metabolic hydrogen, thus limiting substrates for methanogenesis (Henderson, 1980). Soder et al. (2012) showed linear reductions in both CH_4_ production and molar proportion of propionate in continuous culture fermentors dosed with varying levels (0%, 5%, 10%, and 15% of the diet DM) of GFX thereby in agreement with the present results .

We observed a 16% reduction (*P* = 0.055) in enteric CH_4_ production comparing the control diet (0% GFX) with that containing 15% GFX. However, this reduction in enteric CH_4_ production was accompanied by linear decreases in DMI and yields of milk, milk fat, and milk true protein (Resende et al., 2015). Although our results indicate that GFX has moderate enteric CH_4_ mitigation potential (Beauchemin et al., 2020), its negative effects on milk yield and composition are concerning and may limit its use on commercial dairy farms, particularly because producers in the United States do not receive premiums for shipping milk with high concentration of n-3 fatty acids.

### Urinary Purine Derivatives Excretion

Urinary excretion of uric acid, allantoin, and total PD was not affected by diets (Table 2), suggesting that GFX had no impact on microbial protein synthesis in the rumen. Isenberg et al. (2019) reported no differences in the urinary excretion of uric acid, allantoin, and total PD in grazing Jersey cows supplemented (10% of the diet DM) or not with GFX. Furthermore, research done by Soder et al. (2012) in continuous culture fermentors dosed with increasing levels of GFX revealed no change in microbial protein synthesis measured using total purines as the bacterial marker. Even though the urinary excretion of total PD was similar across diets (Table 2), the apparent total-tract digestibilities of organic matter, neutral detergent fiber, and acid detergent fiber all tended to decrease linearly in response to increasing dietary levels of GFX (Resende et al., 2015), thus implying that PUFA released in the rumen possibly affected the growth of fibrolytic microorganisms as discussed earlier. Soder et al. (2012) also reported linear decreases in nutrient digestibility (organic matter and neutral detergent fiber) without a change in the flow of microbial N in continuous culture fermentors dosed with GFX. Collectively , it can be inferred from these results that the lack of GFX effect on microbial protein synthesis in vivo and in vitro may be associated with the growth of certain ruminal microbiota populations compensating for the reduction of other groups resulting in no net gain in microbial mass in the rumen.

**Table 2.** Urinary excretion of purine derivatives (PD) in lactating Jersey cows fed incremental amounts of ground flaxseed (GFX)

Item	GFX, % of the diet DM				SEM	*P*-value^1^		
	0	5	10	15		*L*	*Q*	*C*
Creatinine, m*M*	3.25	3.66	3.31	3.35	0.17	0.95	0.25	0.11
Uric acid, mmol/d	10.3	8.45	9.80	8.00	1.19	0.26	0.98	0.21
Allantoin, mmol/d	137	140	134	122	12.4	0.37	0.55	0.96
Total PD, mmol/d	148	148	144	138	13.2	0.57	0.83	0.95

^1^Probability of linear (*L*), quadratic (*Q*), and cubic (*C*) effects.

In summary, our hypothesis that enteric CH_4_ production would be reduced linearly in response to incremental amounts of GFX due to the toxic effect of GFX-PUFA on growth of methanogens and cellulolytic bacteria was partially confirmed. Enteric CH_4_ production tended to decrease linearly with feeding GFX, but no change was observed in the relative abundance of ruminal archaea genera even though the function of methanogens was not assessed herein. However, linear decreases in the relative abundance of ruminal cellulolytic bacteria genera such as *Ruminococcu*s, *Clostridium*, and *Blautia* may have contributed to the tendency seen for the linear decrease in enteric CH_4_ production in dairy cows fed increasing dietary levels of GFX. Despite a 16% reduction in enteric CH_4_ production with feeding the greatest amount of GFX (i.e., 15% inclusion in the diet DM), yields of milk, milk fat, and milk true protein also decreased by 6.1%, 5.1%, and 9.6%, respectively, as reported in our companion paper (Resende et al., 2015). Therefore, it can be concluded that the tradeoff between the beneficial effect of GFX on mitigating enteric CH_4_ production and its negative effect on yields of milk fat and protein limits the use of GFX in dairy production systems in which producers are paid based on the amount of fat and protein they shipped out of the farm and not on n-3 fatty acid profile or carbon credits .
